# Supplementary material for: Data compression of Bridge Resilience Control: Algorithm and case analysis
Source: PLoS One. 2026 Apr 15;21(4):e0346272. doi: 10.1371/journal.pone.0346272 (PMC13082729; doi:10.1371/journal.pone.0346272)
Supplement: Attachment — Submitted filename: Rebuttal letter.pdf [file pone.0346272.s002.pdf]

Dear Dr Babak Aslani and Reviewers,

Thank you very much for taking the time to review my manuscript entitled " Data Refinement Processing of Bridge Resilience Control Supported by Large-scale model Algorithm and Case Analysis". I greatly appreciate the constructive feedback and insightful comments, which have significantly helped me improve the quality of my work. Below, I provide a point-by-point response to the reviewers' comments. Below, I provide a detailed response to each comment.

Reviewer 1

Comment 1: Literature Review (Section 1.2)

The literature review is too broad and unfocused. Many references (e.g., [8] rumor detection, [12] text-to-image generation, [14] art generation) are tangentially related at best. Reduce this section and focus exclusively on: (a) data processing in structural health monitoring, (b) machine learning in bridge engineering, and (c) time-series compression methods. Clearly identify the research gap your work fills.

Response 1:

According to the reviewer's comments, the Literature Review has been revised by reorganizing the references based on (a) data processing in structural health monitoring, (b) machine learning in bridge engineering, and (c) time series compression methods.

Comment 2: Lack of Validation and Comparison

This is the most serious deficiency. The paper presents NO comparisons with existing methods (PCA, autoencoders, wavelets, SAX, PAA) and uses ONLY synthetic data.

Response 2:

Following the comments from the reviewers, the proposed time-series data compression algorithm is compared with mainstream dimensionality reduction and compression algorithms, including PCA, Auto-Encoders, Wavelet Transform, SAX and PAA. Specifically, the 1D Convolutional Auto-Encoder was employed as the Auto-Encoder method in the comparison. The corresponding comparison results are presented in Fig. 4 of the manuscript.

Comment 3: Methodological Clarity

Several methodological elements need clarification:

3.1 Equation (10): The notation d(1-e^ξ/(1+e^ξ))/d(ξ) is ambiguous. Write it clearly as a derivative.

Algorithm on page 13: "Apply a large-scale model to solve the data loop Dc…" is too vague. Specify the model architecture, training procedure, and loss function.

Parameters like ε=0.05 and window size W need justification through sensitivity analysis.

Response 3:

In accordance with the reviewers' comments, Eq. (10) is revised into the following form. Meanwhile, in Eq. (11), $d_j \rightarrow w_j$ is modified to $d_j(w_j)$.

$$\eta_{ji} = -0.2 \times \frac{d}{d\xi}\left(\frac{1-e^{\xi}}{1+e^{\xi}}\right) \tag{10}$$

$$\xi = 1 - (d_j(w_j)) \tag{11}$$

In the meantime, Equation (9) has been modified to the following form to adopt the differential learning rate.

$$\omega_{ji} = \omega_{ji} - \eta_{ji} x_{ji}(y_i - d_i) \tag{9}$$

In accordance with the reviewers' comments, we have specified which large-scale model to invoke, and revised the expression "Apply a large-scale model to solve the data loop Dc…" to "Apply the neural network model to solve the data loop Dc…". Meanwhile, a brief introduction to the architecture, training procedure, and loss function of the neural network model is added.

In accordance with the reviewers' guidance, the window size is set in a dynamic manner, and the detailed revisions can be found in Section 4.2.1 of the manuscript. In addition, ε has been removed in the revised manuscript, and the value of α is determined according to Eq. (18) in the revised manuscript.

Comment 4: Reproducibility

The statement "available upon reasonable request" is unacceptable for computational work in 2025. Create a public GitHub repository or archive it on Zenodo for a permanent DOI.

Response4:

In accordance with the reviewers' comments, the dataset and source code have been made publicly available.

Comment 5: Results and Analysis

Current results are insufficient:

Figure 1: Only 5 I-beam sections—need at least 50 varied cases

Section 4.2: Only one simple T-beam. Test on continuous spans, cable-stayed, and arch bridges

5% Gaussian noise is unrealistic. Real bridges experience non-Gaussian noise from traffic, wind, and thermal effects。

No analysis of whether 97% compression preserves damage detection capability

Response 5:

In accordance with the reviewers' comments, fifty cross-sections are presented in Fig. 1.

The reviewers suggested conducting tests on different bridge types including continuous span bridges, cable-stayed bridges and arch bridges. We are currently conducting relevant research, and this work will be completed in our subsequent papers.

In accordance with the reviewers' comments, the noise has been modified to 8% non-Gaussian noise (including vehicle noise, wind noise and temperature noise) in the revised manuscript.

In addition, due to the introduction of noise, the compression ratio of the time series data compression algorithm proposed in the revised manuscript is 89.42%. We will analyze the influence of the compressed data on bridge damage detection in subsequent research.

Comment 6:

Terminology Issues

The term "large-scale model" is used inconsistently throughout the manuscript, creating confusion about what type of computational approach is actually being employed. It is often unclear whether you are referring to Large Language Models such as GPT-like systems, deep learning models in general, or simply large neural networks with many parameters. This ambiguity is problematic because these are fundamentally different types of models with different architectures, training paradigms, and appropriate applications. To resolve this issue, you should use standard terminology from the machine learning and structural engineering literature. Specifically, use "deep learning models" when referring to multilayer neural networks, "neural networks" or "feedforward networks" for simpler architectures, and "convolutional networks" or "recurrent networks" when those specific architectures are employed. The term "large language models" or "LLMs" should be reserved exclusively for those instances where you are using transformer-based language models like GPT, BERT, or similar architectures, which appears to occur primarily in your graph classification step. This terminological precision will make your methodology clearer to readers and align your work with standard conventions in both the civil engineering and machine learning communities.

Essential Additions

The manuscript requires several critical additions to meet publication standards. First, you must add a comprehensive section titled "Comparison with State-of-the-Art" that includes a detailed table comparing your method against established techniques. This comparison table should include key metrics as well. A second essential addition is a "Sensitivity Analysis" section that demonstrates the robustness of your proposed method across various conditions. This analysis should systematically vary noise levels different thresholds to show how the algorithm performs under different data quality scenarios. Additionally, you should test the method with missing data percentages ranging from 10% to 70% to establish the limits of your sparse data refinement

approach. The sensitivity analysis must also include different bridge types. Finally, explore how variations in window size W and threshold ε affect the refinement rate and accuracy to guide future users in parameter selection. Third, you must add a "Limitations" section that honestly discusses the constraints and assumptions of your method. This should explicitly acknowledge that the method assumes stationary Gaussian noise and regular sensor grids, which may not hold in all real-world scenarios. You must also acknowledge that validation has been limited to synthetic data and that the method's performance for subtle damage detection in early stages of deterioration remains unknown.

The abstract and contributions sections also require substantial improvement. The abstract should begin by clearly establishing the problem, such as "Bridge monitoring generates massive datasets that challenge current data management and analysis capabilities," before introducing your solution. You must clearly state what is genuinely novel rather than simply stating that you "propose" something. The percentages of 80% and 97% refinement rates need proper context explaining what these numbers mean for practical bridge management. Finally, you must distinguish clearly between your novel contributions, such as the specific application to bridges and the integration of deterioration models into the refinement algorithm, and your adaptations of existing techniques like windowing and interpolation methods.

Language and Presentation

The manuscript requires professional English editing to meet publication standards. Throughout the text, there are numerous instances of awkward phrasing that suggest direct translation from another language. For example, phrases like "with the increase of bridge operation time" should be simplified to "with increased operational time," and expressions such as "cannot be simply integrated" would read more naturally as "cannot be directly unified." Beyond these specific examples, the manuscript suffers from inconsistent mathematical notation, with variables sometimes written as d_i and other times as d_{i,j} without clear explanation of when each form is appropriate, and approximation symbols alternating between ≈ and ≃ apparently interchangeably. These inconsistencies, while seemingly minor, can confuse readers and detract from the

technical content.

Response 6:

In accordance with the reviewers' comments, the term "large model" has been revised to "neural network model" in the revised manuscript, so that readers can clearly understand the method adopted in this study.

The comparison of compression ratios between the time-series data compression algorithm proposed in this paper and the existing algorithms has been supplemented in the revised manuscript. These comparisons are based on the premise that all algorithms adopt the same data fidelity rate (95%).

For missing data compression, the results of data completion for data missing rates ranging from 10% to 70% have been supplemented (Table 1). Meanwhile, taking the 30% data missing rate as an example, the compression ratios corresponding to the initial values of different window sizes have been calculated (Table 2).

In accordance with the reviewers' comments, the limitations of this study have been added to the Conclusions and Future Work section. In particular, it is stated explicitly that the verification is completed based on simulated data, and the actual performance of the proposed algorithms will be further validated in subsequent tests on real bridges.

In accordance with the reviewers' comments, the application value of the compression ratio and data fidelity rate for bridge resilience control has been clearly specified in the abstract.

The expressions and symbols in the manuscript have been revised in accordance with the reviewers' comments.

Reviewer 2

Comment 1:

Please restructure the Introduction according to the standard logic of the research paper; the current logic does not make the theoretical or technical contribution clear.

Response 1:

In accordance with the reviewers' comments, the introduction has been restructured, and the Motivation and Innovation section has been revised.

Comment 2:

Move the literature review currently in Section 1.2 to a new '2. Related Work' section.

Response 2:

In accordance with the reviewers' comments, Section 1.2 has been moved to Section 2 "Related Work".

Comment 3:

There are a large number of inaccurate paragraph divisions in the manuscript. For example, “Correlation refers to the correlation between data. The correlations studied in this article include geometric correlations and temporal correlations. Geometric correlation refers to the geometric shapes or structures composed of data within the subset D_k of a dataset, D_k\subset D. When D_k==D , D_k constitutes the entire bridge structure. Time domain correlation refers to the correlation generated in the time domain of a certain data of a bridge structure over time. For example, the acceleration data and displacement data collected by the bridge health monitoring system all satisfy time-domain correlation.” should be a single paragraph. Please correct all such paragraph divisions throughout the manuscript.

Response 3:

In accordance with the reviewers' comments, the paragraph division of the entire manuscript has been revised in the revised version.

Comment 4:

Provide a nomenclature table that lists every symbol/parameter used in the paper.

Response 4:

A nomenclature of all symbols has been added to **Appendix A** of the revised manuscript.

Comment 5:

All algorithm processes are currently presented in a non-standard format: they lack numbering, explicit Input/Output blocks, etc. In addition to presenting the algorithms in the standard way, it is recommended to use more descriptive language for the algorithm processes. For example, using '→' directly in an algorithm without explanation can confuse readers (in mathematical expression, '→' can mean 'tends to'; in pseudocode, it is often understood as 'assignment').

Response 5:

In the revised manuscript, we have presented the original algorithm in the form of pseudocode to enable readers to understand it more clearly.

Comment 6:

Supply pseudocode for every algorithm, either in the main text or in an appendix.

Response 6:

In accordance with the reviewers' requirements, we have provided the pseudocode of the algorithm in the revised manuscript.

Comment 7:

Step 7 of the time domain data refinement algorithm states 'Call the large-scale model to classify G and generate a classification quantity k'. What the large-scale model is used in this step? What is the specific classification algorithm? How are the specific parameters set? If these issues are not clearly stated in the manuscript, the scientificity and accuracy of the subsequent analysis results cannot be guaranteed.

Response 7:

In Step 7, the DeepSeek-chat model is invoked to classify the graphs generated by the curvature mode. When the number of categories $X = 1$, the data in this category is compressed by taking the mean value of the data within the category. When the number of categories $X > 1$, half of the window size is used to perform reclassification. Meanwhile, the data fidelity rate is set to 95% in the program design. In the revised manuscript, the large model is specified as DeepSeek-chat.

Comment 8:

The claim 'The resilience-control data of bridges is affected by factors such as the number of equipment and equipment failures' is unconvincing as an explanation for data sparsity. Commercial sensing systems are designed to fail rarely; therefore, device failure alone cannot justify the observed scarcity. Provide stronger evidence or cite published studies that substantiate this argument.

Response 8:

In response to the reviewers' comments, we have cited references [24, 25, 26] in the revised manuscript to demonstrate that the sparse nature of the bridge resilience control data arises from factors such as the number of devices and device failures.

Comment 9:

Justify the choice of ε=0.05 in Equation (18).

Response 9:

In the revised manuscript, ε has been removed in the revised manuscript, and the value of α is determined according to Eq. (18) in the revised manuscript.

Comment 10:

Compare the proposed refinement algorithms with state-of-the-art alternatives to demonstrate their superiority.

Response 10:

Following the comments from the reviewers, the proposed time-series data compression algorithm is compared with mainstream dimensionality reduction and compression algorithms, including PCA, Auto-Encoders, Wavelet Transform, SAX and PAA. Specifically, the 1D Convolutional Auto-Encoder was employed as the Auto-Encoder method in the comparison. The corresponding comparison results are presented in Fig. 4 of the manuscript.

Reviewer 3

Comment 1:

Article Title

The title is very ok and it flows along just that it is too long the author should find a way to refine it a bit.

Response 1:

In accordance with the reviewers' comments, the title of the paper has been revised to Data compression of Bridge Resilience Control: Algorithm and Case Analysis.

Comment 2:

Abstract

The abstract is good to go; everything is stated correctly, from its introduction to the problem, methodology, and result output was mentioned as related to its simulation analysis but it does not mention its performance explicitly rather it proceeded to the future research.

It is also advisable to use a grammar editor or an expert in grammar or language to assist in correcting grammatical and punctuation errors.

Response 2:

Several revisions have been made in accordance with the reviewers' comments, as follows: First, the shortcomings of existing data compression algorithms have been added to the abstract. Second, it has been clarified in the abstract that the algorithm proposed in this paper can reduce the data storage cost and transmission bandwidth consumption, and the feature retention accuracy can meet the requirements for bridge resilience control. Third, the erroneous grammar and punctuation in the manuscript have been corrected.

Comment 3:

LITERATURE REVIEW

The review is done, properly referenced, concurrence is important as it is supposed

to flow along and tailored to the current research which means that the closest research need to be considered.

Response 3:

In accordance with the reviewers' comments, the Literature Review has been rewritten and designated as Section 2 "Related Work" of the manuscript.

Comment 4:

PERFOMANCE METRICS

Algorithms are well and properly stated, the system model and the transformation of time domain data is well articulated.

Response 4:

The descriptions of the algorithms have been further revised, and all algorithms are presented in the form of pseudocode in the revised manuscript.

Comment 5:

METHODOLOGY:

- This is properly presented, there are a lot of information mentioned in the methods and strategy which has been explicitly described.
- Kindly re-arrange the Methodology for simplicity and easy tracking so that It can be simpler to relate the methods, results and output.

Response 5:

The reviewers' comments have been fully adopted, and the research methodology is simplified in the revised manuscript with three main revisions: (1) The DeepSeek-chat model is explicitly adopted for domain knowledge-based data compression; (2) For time-series data compression, the large model is clearly defined as a neural network model; (3) New figures and tables are incorporated into the simulation analysis for clearer presentation of the simulation results.

Comment 6

RESULT

- The result is well presented inclusive of all necessary graph.
- The concluding part is too scanty compare to too many equations and solutions provided, I will suggest it should be a more explicit way of concluding
- Supporting information is not necessary, but it can also be integrated into the conclusion in a summarized manner.

Response 6:

In accordance with the reviewers' comments, the Conclusions section has been rewritten in the revised manuscript. The conclusion has been supplemented with the finding that the algorithms proposed in this study can improve the data compression ratio while preserving the physical information of bridge structures, and the compressed data has clear physical meaning. Meanwhile, the limitations of this study have also been analyzed in the Conclusions and Future Work section.

Comment 7:

CONCLUSION:

This paper is good as it stated the key aspect of the research and it widen the scope as it continue to state explicitly the research motivation, and it is very important in the core area of Algorithm and Case Analysis so it requires a lot of attention while dissemination them.

Response 7:

We would like to thank the reviewers for their valuable guidance. These comments have greatly improved the quality of this manuscript. We will address the limitations of this study in future research to enhance the practicality of the research outcomes in bridge resilience control.

Sincerely,

Ming Chen

Shanghai ZhongQiao Vocational and Technical University

chenmchen1975@126.com
