# Supplementary material for: Data compression of Bridge Resilience Control: Algorithm and case analysis
Source: PLoS One. 2026 Apr 15;21(4):e0346272. doi: 10.1371/journal.pone.0346272 (PMC13082729; doi:10.1371/journal.pone.0346272)
Supplement: Attachment — Submitted filename: Rebuttal letter-2.pdf [file pone.0346272.s003.pdf]

Dear Dr Babak Aslani and Reviewers,

Thank you very much for taking the time to review my manuscript entitled " Data Refinement Processing of Bridge Resilience Control Supported by Large-scale model Algorithm and Case Analysis". I greatly appreciate the constructive feedback and insightful comments, which have significantly helped me improve the quality of my work. Below, I provide a point-by-point response to the reviewers' comments.

Reviewer 2

Comment:

The revised manuscript presents its innovation and technical details more clearly, but also exposes potential technical risks. The DeepSeek-chat model, a famous LLM, is invoked to classify the graphs generated by the curvature mod. However, LLM is probability generated. Its primary goal is to generate natural, diverse, and creative text, rather than conducting stable deterministic calculations. For tasks such as graph classification that require structural awareness and deterministic computation, specialized GNN models far outperform LLM in accuracy, efficiency, consistency, and interpretability. LLM is more suitable as an auxiliary tool for interpreting, summarizing, or generating descriptive text based on graph data. Therefore, I believe that there is technical uncertainty in this work, which may lead to potential technical risks in the actual operation process. My final suggestion is to reject the manuscript.

In addition, there is a small issue that the author can refer to: the next paragraph of equation (15) repeats the discussion after equation (14).

Response :

I sincerely appreciate the insightful and constructive comments raised by the reviewer, which have identified a key technical limitation in my manuscript and provided valuable guidance for enhancing the rigor and reliability of the research. We fully recognize and accept the core viewpoints of the reviewer. In the revised manuscript, I have adopted the GIN (Graph Isomorphism Network) to judge the similarity between graphs, and revised the algorithm process and corresponding results accordingly. Meanwhile, the redundant content in the paragraphs following Formula (15) has been

deleted.

Sincerely,

Ming Chen

Shanghai ZhongQiao Vocational and Technical University

chenmchen1975@126.com
